# Heat-Related Emergencies in the Gulf Region: A Literature Review of the Current Epidemiology, Diagnosis, and Effective Management Strategies

**DOI:** 10.7759/cureus.113267

**Published:** 2026-07-23

**Authors:** Basmalah Naji, Salim Fredericks

**Affiliations:** 1 Medicine, Royal College of Surgeons in Ireland, Muharraq, BHR; 2 Biochemistry, Royal College of Surgeons in Ireland, Muharraq, BHR

**Keywords:** gulf cooperation council, gulf region, heat illness, high temperatures, management

## Abstract

Heat-related emergencies constitute a major and growing public health challenge in the Gulf region due to extreme climatic conditions, rapid urbanization, and widespread occupational heat exposure. Rising ambient temperatures, high humidity, and recurrent heatwaves significantly increase the incidence of heat-related illnesses, particularly heat exhaustion and heatstroke, placing substantial strain on emergency healthcare services. This literature review aims to synthesize existing evidence on the epidemiology, environmental and occupational risk factors, pathophysiology, clinical presentation, diagnostic challenges, and management strategies of heat-related emergencies in Gulf Cooperation Council (GCC) countries. A structured search of PubMed, Scopus, and Google Scholar was conducted to identify relevant studies published between 2010 and 2025. The findings indicate that heat-related illnesses disproportionately affect outdoor workers, pilgrims, and vulnerable populations such as older adults and individuals with chronic disease. Delayed presentation, diagnostic overlap with other acute conditions, and variability in emergency department cooling practices contribute to adverse outcomes, including multiorgan dysfunction and increased mortality. Early recognition and rapid initiation of effective cooling remain the cornerstone of management. Preventive strategies, including occupational heat regulations, public health interventions, and early warning systems, are essential to mitigate the escalating burden of heat-related emergencies in the region. Strengthening standardized clinical protocols and region-specific prevention policies is critical in the context of ongoing climate change.

## Introduction and background

In the height of summer, temperatures across the Gulf can soar beyond 50°C, a level of heat that the human body was never designed to endure. In recent decades, the Gulf region has become one of the fastest-warming areas on the planet, with heatwaves growing more frequent, more intense, and more deadly. Climate projections warn that parts of the Arabian Peninsula may face "unprecedented heat extremes" capable of surpassing human physiological tolerance by the end of the century. Under these conditions, a simple day outdoors can become life-threatening within minutes [1].

Heat-related emergencies have therefore shifted from being rare, seasonal events to a persistent public health concern in countries such as Saudi Arabia, the United Arab Emirates (UAE), Bahrain, Kuwait, Qatar, and Oman. These nations record some of the highest global wet-bulb temperatures, which are the lowest temperatures to which air can be cooled by the evaporation of water. This is a combined measure of heat and humidity that reflects true heat stress on the body. When wet-bulb temperatures exceed 35 degrees, even a healthy adult sitting in the shade can't maintain a safe core body temperature. Alarmingly, such conditions have already been documented in parts of the Gulf [2,3].

The consequences are evident in the region's emergency departments (EDs), where cases of heat exhaustion, heatstroke, dehydration, rhabdomyolysis, and acute renal injury rise sharply during the summer months. Exertional heatstroke, typically associated with intense physical activity under extreme heat, is especially common among the Gulf's large population of migrant laborers, who often work long hours outdoors with limited access to shade, cooling, or hydration. Studies from the UAE and Qatar show that outdoor workers represent a disproportionate share of heat-related hospitalizations and that delayed cooling and lack of early recognition significantly worsen clinical outcomes [4-6]. Yet the burden extends beyond laborers. Elderly individuals, those with chronic illnesses, pilgrims undertaking physically demanding rituals during the Hajj season, and athletes exposed to intense training are all at heightened risk. Research from Saudi Arabia has documented significant spikes in heat-related morbidity during Hajj, particularly when rituals coincide with hotter months. Despite this, gaps in public awareness, inconsistent implementation of preventive policies, and limited access to immediate cooling resources contribute to missed diagnoses and preventable complications [7,8].

Heatstroke, the most severe form of heat illness, is a medical emergency defined by a core temperature typically above 40°C and central nervous system dysfunction. Without rapid cooling, ideally within the first 30 minutes, mortality rates can exceed 80%. The urgency of timely management underscores the need to understand how heat emergencies present in the Gulf, what factors hinder prompt diagnosis, and which strategies effectively reduce morbidity and mortality in this unique climatic environment [9,10].

Given the increasing severity of climate conditions in the Gulf and the substantial human and economic burden associated with heat-related emergencies, a comprehensive synthesis of current evidence is essential. This literature review, therefore, examines the epidemiology, clinical features, diagnostic challenges, management practices, and preventive strategies surrounding heat-related emergencies in the Gulf region while identifying gaps that require further research. 

## Review

Methods 

A structured literature review was conducted to summarize the current evidence on heat-related emergencies in the Gulf Cooperation Council (GCC) countries. Relevant literature was identified through searches of PubMed, Scopus, and Google Scholar for articles published between January 2010 and March 2025. The search strategy used combinations of keywords including "heat-related illness," "heatstroke," "heat stress," and "hyperthermia," together with regional terms such as "Gulf region," "GCC," "Saudi Arabia," "United Arab Emirates," "Qatar," "Kuwait," "Oman," and "Bahrain." The review focused on peer-reviewed studies involving human participants conducted within GCC countries that reported on the epidemiology, risk factors, clinical manifestations, diagnosis, management, or prevention of heat-related illnesses (HRIs). Animal studies, laboratory-based investigations, conference abstracts, editorials, and studies conducted outside the GCC region were excluded. Information relevant to the objectives of the review, including study characteristics, clinical findings, diagnostic approaches, management strategies, and preventive measures, was extracted. The findings were synthesized narratively to summarize the current literature on heat-related emergencies in the GCC region.

Epidemiology of heat-related emergencies in the Gulf region 

HRIs, including heat exhaustion, heatstroke, dehydration, and heat stress-associated complications, represent an escalating public health concern across the GCC countries. Within this extreme climate context, epidemiological data from GCC countries demonstrate a marked seasonal rise in heat-related ED visits and hospital admissions. These environmental conditions have transformed heat-related emergencies from sporadic seasonal events into predictable and recurrent health threats with substantial clinical and economic consequences. Evidence suggests that the true burden of HRIs is likely underestimated due to underreporting, limited surveillance systems, and misclassification of heat-related deaths and hospitalizations [3].

Epidemiological data consistently demonstrate a marked seasonal pattern in heat-related emergencies across the Gulf, with a sharp rise in ED visits and hospital admissions during the summer months, typically from May through September. During this period, sustained thermal stress places significant strain on thermoregulatory mechanisms, increasing the risk of heat exhaustion and progression to heatstroke. Unlike temperate regions, where heat illness is often linked to short-lived heatwaves, Gulf countries experience prolonged exposure to extreme heat, leading to cumulative physiological stress and a higher baseline risk throughout the summer season. A scoping review examining heat-related health outcomes in the Arabian Peninsula identified a growing body of evidence linking extreme temperatures to increased morbidity and mortality, particularly during sustained heat periods [3].

Humidity further amplifies heat risk in many Gulf states, especially in coastal areas such as the UAE, Qatar, and Bahrain. Elevated wet-bulb temperatures, which reflect the combined effects of heat and humidity on the body's ability to dissipate heat, are increasingly recognized as a critical determinant of HRIs. Climate studies have demonstrated that parts of the Arabian Peninsula already experience wet-bulb temperatures approaching dangerous physiological thresholds, beyond which even healthy individuals can't maintain thermal homeostasis without external cooling. These conditions are strongly associated with increased rates of heat-related hospitalizations and adverse outcomes [3].

Country-specific epidemiological patterns highlight notable regional differences in exposure and risk. Saudi Arabia bears a particularly high burden due to its geographic size, desert climate, and the annual influx of millions of pilgrims during Hajj. When pilgrimage seasons coincide with peak summer temperatures, large spikes in HRIs are observed. The extreme heat experienced during the 2024 Hajj, when temperatures exceeded 50°C, resulted in thousands of documented heat-related cases and highlighted the ongoing vulnerability of mass gatherings to heat emergencies [11]. Despite extensive preventive measures, heatstroke remains a leading cause of morbidity during such events.

In the UAE and Qatar, rapid urbanization combined with a large outdoor workforce contributes to significant heat exposure. EDs in both countries report seasonal surges in heat exhaustion, dehydration, and exertional heatstroke during prolonged heatwaves. These clinical patterns align with rising summer temperatures and increased frequency of extreme heat days documented across the region [12]. In Kuwait, epidemiological studies have demonstrated a strong association between extreme heat days and increased occupational injuries and heat-related health events, even in the presence of midday work bans, suggesting that existing protective policies may be insufficient under escalating climate conditions [6].

Across all GCC countries, certain demographic groups are disproportionately affected by heat-related emergencies. Migrant and outdoor workers account for a disproportionate share of heat-related hospital admissions and mortality during summer months. Studies and reports indicate that migrant workers experience substantially higher risks of heat-related morbidity and mortality compared to the general population, reflecting structural vulnerabilities and occupational exposure [13].

Additional high-risk groups include athletes, military personnel, and individuals engaging in strenuous outdoor physical activity, particularly when activity coincides with peak environmental heat [14]. 

**Figure 1:**
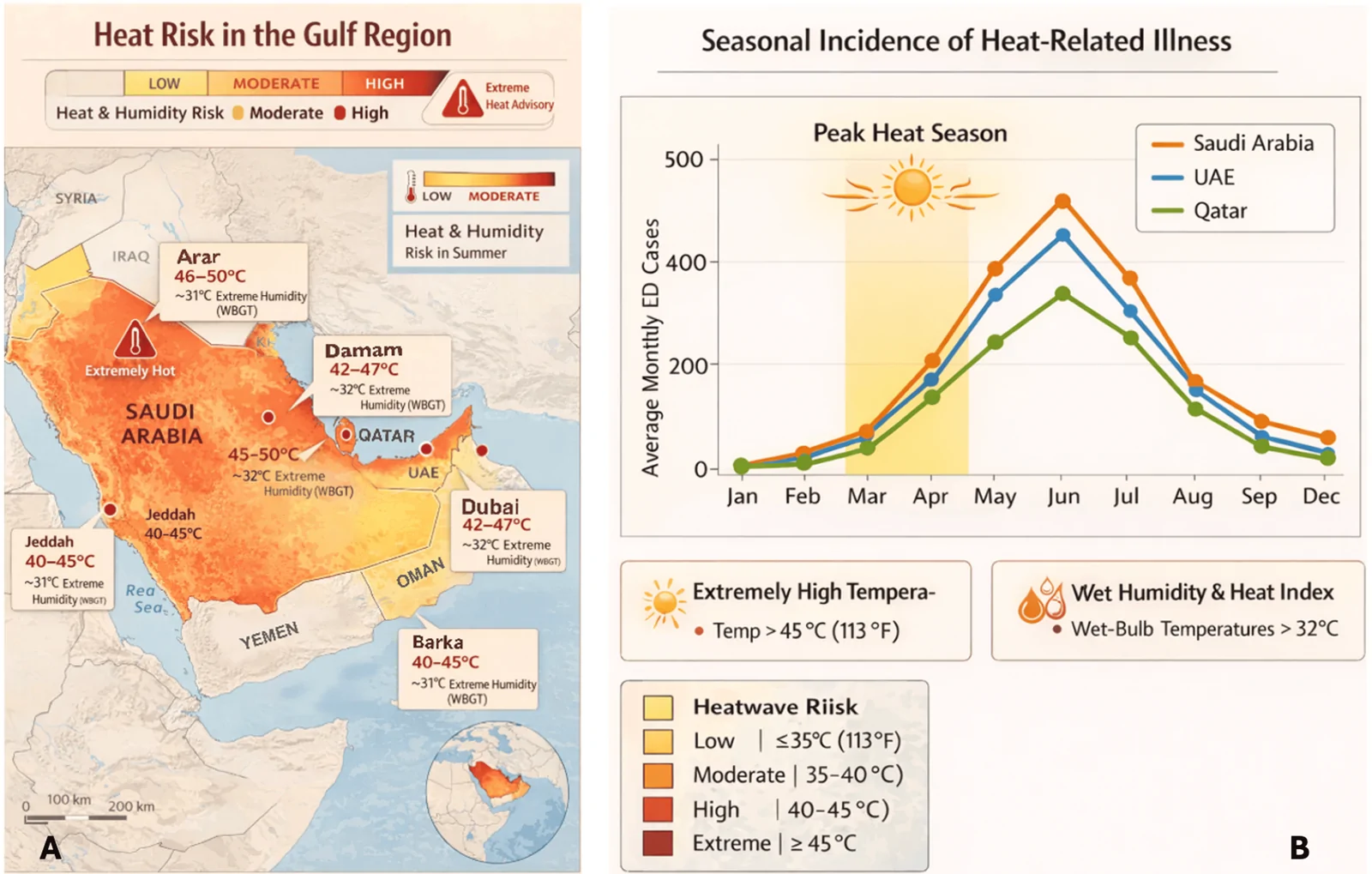
Heat-related risk and seasonal incidence of heat-related illness in the Gulf Cooperation Council (GCC) region. (A) Geographic distribution of heat-related risk across GCC countries, highlighting areas with high ambient temperatures and elevated wet-bulb globe temperature (WBGT) during the summer months. (B) Schematic representation of the seasonal trend in heat-related emergency department presentations, demonstrating the increase in cases during the peak heat season (May–July). Created by the authors based on published literature. Original figure created by the authors. Panels A and B were manually illustrated using Sketchbook (Sketchbook, Inc., San Francisco, California) on an iPad, and the labels and text formatting were added using Canva (Canva Pty Ltd., Sydney, Australia). The arrangement and layout were developed by the authors, with minor AI assistance used to conceptualize the organization of the figure.

Figure 1A illustrates the GCC countries, including Saudi Arabia, the UAE, Qatar, Kuwait, Oman, and Bahrain, highlighting areas exposed to extreme summer temperatures and high humidity. Color gradients represent increasing levels of heat stress, reflecting regions with elevated ambient temperatures and wet-bulb values that impair human thermoregulation. The figure emphasizes the geographic concentration of high heat risk across the Gulf, particularly in densely populated urban and industrial zones, underscoring the environmental context contributing to the high burden of heat-related emergencies in the region. Figure 1B shows trends in heat-related ED cases across Gulf countries. While nationally standardized ED data are limited, studies from Saudi Arabia report significant proportions of heat-related hospital admissions during peak summer and Hajj seasons, with heat exhaustion and heatstroke comprising the major diagnostic categories. Reports from the UAE indicate increases in ED presentations for heat exhaustion and dehydration during extreme heat periods, and retrospective data from Qatar show hundreds of heat fatigue cases during hot months [15,16].

Environmental and occupational risk factors 

In the Gulf region, a constellation of environmental and occupational risk factors dramatically escalates the likelihood of HRIs, particularly during the prolonged hot season. Extreme temperature exposure combined with high humidity creates conditions in which the human body struggles to regulate heat. Unlike arid heat alone, humid heat significantly impairs the body's ability to dissipate heat through sweating, meaning that even healthy individuals can rapidly accumulate dangerous internal temperatures under sustained exposure. This combined stress of high air temperature and humidity is a defining characteristic of Gulf climates during summer and a key driver of heat illness risk among exposed populations [17].

Urban centers across the Arabian Peninsula also amplify environmental heat exposure through the urban heat island (UHI) effect. UHI arises when densely built environments composed of dark, impermeable surfaces such as asphalt and concrete absorb and re-radiate solar energy, elevating local temperatures compared with surrounding rural areas. The reduction of green spaces further limits natural cooling from evapotranspiration, exacerbating human thermal discomfort and occupational heat stress in cities such as Riyadh, Dubai, and Doha. These urban microclimates can elevate day-to-day exposures beyond regional ambient conditions, contributing to both community heat burden and occupational risk in populated urban zones [18].

Outdoor workers in the Gulf face some of the highest occupational heat exposures globally. Construction laborers, oil-field workers, delivery personnel, and others engaged in prolonged outdoor work are regularly exposed to daytime temperatures often exceeding 40°C-45°C, frequently with high humidity that elevates physiological heat strain. Environmental monitoring studies conducted at construction sites in Saudi Arabia's Al-Ahsa province found that heat stress indices such as the wet-bulb globe temperature (WBGT) regularly exceeded recommended safety thresholds throughout the workday, exposing workers to significant heat stress risk even outside the midday peak heat hours. These conditions persisted despite existing regulated outdoor work breaks, underscoring how extreme environmental heat can overwhelm both natural and administrative protections. In addition to acute heat illness, sustained environmental heat exposure has been linked to subclinical health effects among workers, such as acute kidney injury, which is associated with dehydration and repeated heat stress over the work season [5,17].

Interlinked with environmental heat is the intensity of physical labor itself. Outdoor work often requires strenuous physical exertion, which increases internal heat production, further compounding the challenges of external heat exposure. Workers engaged in heavy manual tasks exhibit elevated heart rates, increased core heat load, and faster fluid loss, which, if not offset by sufficient rest, hydration, and cooling opportunities, greatly escalates the risk of heat exhaustion, heatstroke, and longer-term organ stress. Prolonged exposure to such conditions without adequate mitigation has been repeatedly identified as a major risk factor for occupational injuries and HRIs in the Gulf [5].

Beyond routine occupational exposure, high-intensity events such as the Hajj pilgrimage and competitive sports events create episodic yet profound heat stress risks. During Hajj, one of the largest annual mass gatherings in the world, millions of pilgrims perform physically demanding rituals outdoors, often in periods of extreme heat. Heat load during crowded rituals is amplified not only by high ambient temperatures but also by body heat from surrounding crowds and by restricted airflow in temporary housing settings, thereby increasing physiological strain and the risk of heat emergencies among aging or physically vulnerable pilgrims [19].

Risk factors also intersect with clothing, hydration barriers, and cultural constraints that influence individuals' responses to heat. Traditional attire in many Gulf societies, while culturally significant and protective against solar radiation under some conditions, can limit evaporation of sweat and restrict airflow over the skin, reducing the body's capacity for heat dissipation when humidity is high. Hydration practices may also be constrained by work schedules, cultural norms, and limited access to cool drinking water in field settings. In occupational environments where work pace is driven by productivity requirements or where workers fear job loss for taking breaks, opportunities for adequate hydration and rest are often insufficient, increasing susceptibility to heat stress and dehydration [20].

Pathophysiology of heat illness 

Heat illness arises when the body's finely tuned thermoregulatory mechanisms fail to maintain a safe internal temperature in the face of excessive environmental heat, physical exertion, or both. Under normal conditions, human heat balance depends on the balance between heat production from metabolism and muscle activity and heat loss through radiation, conduction, convection, and, most critically in hot conditions, the evaporation of sweat from the skin and respiratory surfaces. As ambient temperature rises above body temperature, evaporative cooling becomes the principal mechanism for heat dissipation. When this system is overwhelmed, either because sweat evaporation is impeded by high humidity or because heat production outpaces heat loss, core temperature begins to rise. As the core temperature increases, cutaneous vasodilation and sweating initially help to dissipate heat by increasing blood flow to the skin and facilitating evaporative cooling. However, these compensatory responses also reduce blood flow to internal organs such as the kidneys and gastrointestinal tract, contributing to hypoperfusion and organ stress, while dehydration progressively compounds circulatory compromise. Ultimately, when heat gain exceeds the body's capacity to lose heat, thermoregulatory failure ensues, and systemic heat injury occurs [21,22].

The progression of HRIs generally follows a continuum from heat exhaustion to heatstroke. Heat exhaustion represents an early, less severe stage characterized by fatigue, dizziness, weakness, nausea, and often a core body temperature that remains below the threshold for heatstroke. If heat stress continues unchecked and core temperature rises above approximately 40°C, the result can be heatstroke, a life-threatening syndrome defined by severe hyperthermia and central nervous system dysfunction such as confusion, delirium, seizures, or coma. At this stage, the body's thermoregulatory mechanisms are no longer effective, and clinical signs of systemic involvement rapidly develop [21,23].

At the cellular and systemic levels, heatstroke triggers a complex pathophysiological cascade involving direct thermal injury, oxidative stress, inflammatory activation, and coagulopathy. High core temperatures denature cellular proteins and disrupt membrane integrity, leading to cellular dysfunction and release of damage-associated molecular patterns (DAMPs) that stimulate innate immune responses. This, in turn, activates macrophages and monocytes to release pro-inflammatory cytokines, such as interleukin-6 and tumor necrosis factor-α, contributing to a systemic inflammatory response syndrome (SIRS) that resembles sepsis. Endothelial cell injury, neutrophil activation, and neutrophil extracellular trap (NET) formation further promote microvascular thrombosis. Concurrently, alterations in coagulation pathways and suppressed fibrinolysis can lead to heatstroke-associated coagulopathy, which in severe cases may evolve into disseminated intravascular coagulation (DIC) with widespread microthrombi and bleeding. These processes collectively contribute to multiorgan dysfunction affecting the central nervous system, kidneys (often resulting in acute kidney injury), liver, lungs, and heart [24,25].

Inflammation and coagulopathy are central drivers of the severity of heatstroke. As the systemic inflammatory response intensifies, increased vascular permeability and endothelial dysfunction further impair organ perfusion, compounding heat-related tissue damage. Rhabdomyolysis, the breakdown of skeletal muscle, frequently accompanies severe hyperthermia, especially in exertional heatstroke, liberating myoglobin into the circulation and increasing the risk of renal injury and electrolyte abnormalities. Without rapid and effective cooling, these pathophysiological processes can culminate in shock, multiorgan failure, and high mortality [26].

Classically, heatstroke is categorized into two main patterns: classic (non-exertional) heatstroke and exertional heatstroke, each with distinct triggers and clinical contexts that are especially relevant to the Gulf region. Classic heatstroke typically develops over hours to days of passive exposure to extreme environmental heat, often affecting vulnerable populations such as the elderly, those with chronic disease, and individuals with limited access to cooling and hydration. In contrast, exertional heatstroke occurs rapidly in young, healthy, and physically active individuals during strenuous activity in hot conditions. In exertional heatstroke, the internal heat generated by intense exertion, combined with environmental heat, overwhelms thermoregulatory capacity, leading to abrupt, severe hyperthermia and a higher likelihood of complications such as rhabdomyolysis and acute kidney injury. Although both forms share core features-severe hyperthermia and central nervous system dysfunction-they differ in their onset, affected populations, and some clinical manifestations, with exertional heatstroke often occurring in the context of physical activity such as sports, military training, or labor in hot environments [21,27]. 

**Figure 2:**
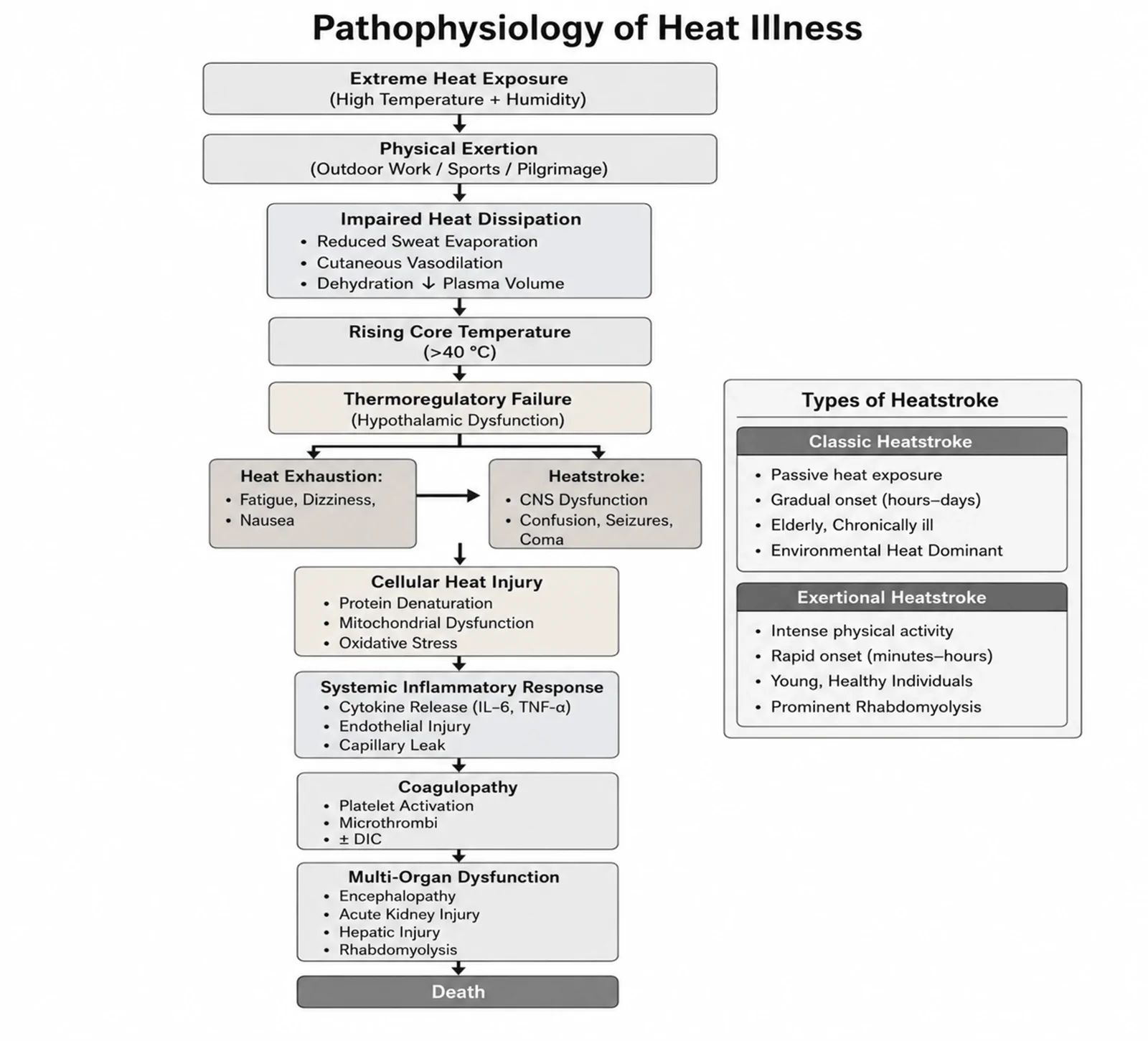
Cellular and systemic pathophysiological mechanisms involved in heat stroke, including thermoregulatory failure, inflammatory activation, endothelial dysfunction, and multiorgan injury. Original figure created by the authors.

Figure 2 illustrates the pathophysiological cascade of HRIs, beginning with excessive environmental or exertional heat exposure and failure of normal thermoregulatory mechanisms. Impaired heat dissipation leads to progressive hyperthermia, which in turn causes cellular injury, protein denaturation, and mitochondrial dysfunction. Subsequent systemic inflammatory activation, endothelial damage, and coagulation abnormalities contribute to microvascular impairment and tissue hypoperfusion. These processes culminate in multiorgan dysfunction, including central nervous system injury, rhabdomyolysis, acute kidney injury, hepatic failure, and cardiovascular instability [28].

Clinical presentation and diagnostic challenges 

HRIs present a spectrum of clinical manifestations, with the severity of symptoms reflecting the underlying degree of thermoregulatory failure and systemic involvement. The hallmark clinical features of heatstroke, the most severe form of heat illness, include hyperthermia, defined as a core body temperature typically exceeding 40°C, and central nervous system (CNS) dysfunction, which can manifest as confusion, delirium, seizures, or even coma. Accompanying signs often include tachycardia, hypotension, dizziness, nausea, vomiting, and varying degrees of dehydration due to substantial fluid loss from excessive sweating and inadequate intake. Patients may also present with altered skin perfusion, ranging from warm and flushed in the early stages to cool and clammy in cases of circulatory compromise. In less severe heat illnesses, such as heat exhaustion, the primary features include fatigue, weakness, and mild cognitive impairment, though these symptoms can rapidly progress if heat exposure continues unchecked [21]. 

Table 1 compares and highlights the main differences between heat exhaustion and heatstroke while also outlining their management. Heat exhaustion is characterized by symptoms such as dizziness, thirst, heavy sweating, nausea, and weakness, and is depicted as a potentially reversible condition with early intervention, including moving to a cooler environment, loosening clothing, and oral hydration. In contrast, heatstroke is illustrated as a life-threatening emergency marked by central nervous system dysfunction, including confusion, dizziness, and loss of consciousness. The figure emphasizes the need for urgent medical intervention in heatstroke, highlighting rapid cooling measures and emergency services activation [29,30].

**Table 1:** A comparison of the difference between heat exhaustion and heatstroke.

Feature	Heat Exhaustion	Heat Stroke
Severity and core body temperature	Mild to moderate, core body normal or mildly elevated (<40°C)	Severe, life-threatening emergency. Core body (>40°C)
Mental status	Normal or mild confusion	Altered mental status, delirium, seizures, coma
Skin	Pale, cool, clammy	Hot, flushed, may be dry or sweaty
Heart rate and blood pressure	Tachycardia, normal/orthostatic hypotension	Marked tachycardia, hypotension, shock
Symptoms	Fatigue, dizziness, nausea, headache, muscle cramps [29]	Severe headache, confusion, collapse, seizures [29]
Management	Move to a cooler area, sip cool water, seek medical help if symptoms don't improve [30]	Call emergency service immediately; loosen clothing and cool with water and ice; Immediate cooling and ICU care [30]

Laboratory evaluation plays a critical role in assessing the severity of HRIs and guiding management. Elevated creatine kinase (CK) levels are frequently observed, particularly in exertional heatstroke, reflecting rhabdomyolysis and the associated risk of acute kidney injury. Electrolyte disturbances, such as hypernatremia, hyponatremia, hypokalemia, or hyperkalemia, are common and arise from fluid shifts, dehydration, and renal dysfunction. Assessment of renal function via serum creatinine and blood urea nitrogen (BUN) is essential for detecting acute kidney injury early. Coagulopathy, ranging from mild thrombocytopenia to full DIC, can also occur due to endothelial injury and systemic inflammatory response. Liver function tests may show mild to moderate elevations, while lactate and other markers of tissue hypoperfusion can indicate systemic compromise. These laboratory findings not only confirm the diagnosis but also help identify complications that require urgent intervention [26].

In emergency settings, diagnostic tools primarily focus on rapid clinical assessment and supportive investigations. Core body temperature measurement, ideally via rectal thermometry, remains the gold standard for identifying severe hyperthermia. Continuous monitoring of vital signs, including heart rate, blood pressure, respiratory rate, and oxygen saturation, allows clinicians to track physiologic derangements and guide resuscitation. Point-of-care testing, including rapid blood chemistry panels, CK, and coagulation studies, facilitates timely recognition of complications such as rhabdomyolysis, electrolyte imbalances, and coagulopathy. Imaging is generally reserved for patients with suspected complications such as stroke, seizures, or organ injury, rather than for initial diagnosis [21].

In the Gulf region, diagnostic challenges are amplified by unique environmental and social factors. Delayed presentation to healthcare facilities is common, particularly among migrant workers, due to factors including limited awareness of heat illness, fear of employment consequences, and logistical barriers to accessing care. The lack of immediate cooling resources at work sites or in community settings further exacerbates the risk of rapid progression from heat exhaustion to heatstroke. High patient volumes during peak summer periods can overwhelm EDs, reducing the time available for thorough assessment. Additionally, communication barriers, including language differences and varying literacy levels among migrant populations, can hinder accurate history-taking and symptom reporting. Cultural and social factors, such as reluctance to report early symptoms or limited understanding of occupational safety protocols, further complicate timely recognition and intervention. Collectively, these challenges contribute to delayed diagnosis, increased complication rates, and higher morbidity and mortality in vulnerable populations within the Gulf region [17].

ED management strategies 

Management of heat-related emergencies in the ED centers on rapid recognition, immediate cooling, hemodynamic stabilization, and prevention of secondary complications. Immediate cooling is the most critical intervention for heatstroke, as mortality rises sharply with each minute of delayed temperature reduction. Preferred cooling methods include evaporative cooling, spraying patients with tepid water while fanning to enhance heat loss, and ice-water immersion, which is particularly effective for exertional heatstroke. In situations where immersion is not feasible, alternative methods such as cold-water dousing, cooling blankets, or ice packs applied to major vascular areas (neck, axillae, groin) are recommended. Core temperature should be monitored continuously to guide therapy, and cooling efforts are typically maintained until the body temperature falls below 38°C-39°C to prevent overshoot hypothermia [21].

Fluid resuscitation is another cornerstone of ED management, aiming to restore intravascular volume, correct dehydration, and support end-organ perfusion. Isotonic crystalloids, such as 0.9% saline, are preferred and administered rapidly, especially in patients with hypotension or signs of hypoperfusion. Electrolyte imbalances, frequently observed in heat-related emergencies, should be monitored and corrected accordingly. In cases of significant rhabdomyolysis or hypernatremia, fluid therapy may need to be adjusted carefully to prevent complications such as fluid overload or worsening electrolyte derangements. Continuous cardiac and renal monitoring is recommended for patients at risk of acute kidney injury or arrhythmias [26].

Patients presenting with severe heatstroke require vigilant monitoring for complications, including rhabdomyolysis, acute kidney injury, liver injury, electrolyte disturbances, and coagulopathy. Laboratory assessment typically includes serial measurements of CK, renal function markers, liver enzymes, electrolytes, and coagulation parameters. Early recognition of these complications allows timely interventions such as aggressive hydration, renal replacement therapy if indicated, and supportive care for systemic organ dysfunction. Multidisciplinary involvement, including nephrology and critical care teams, may be required for patients with severe or multiorgan involvement [24]. 

**Figure 3:**
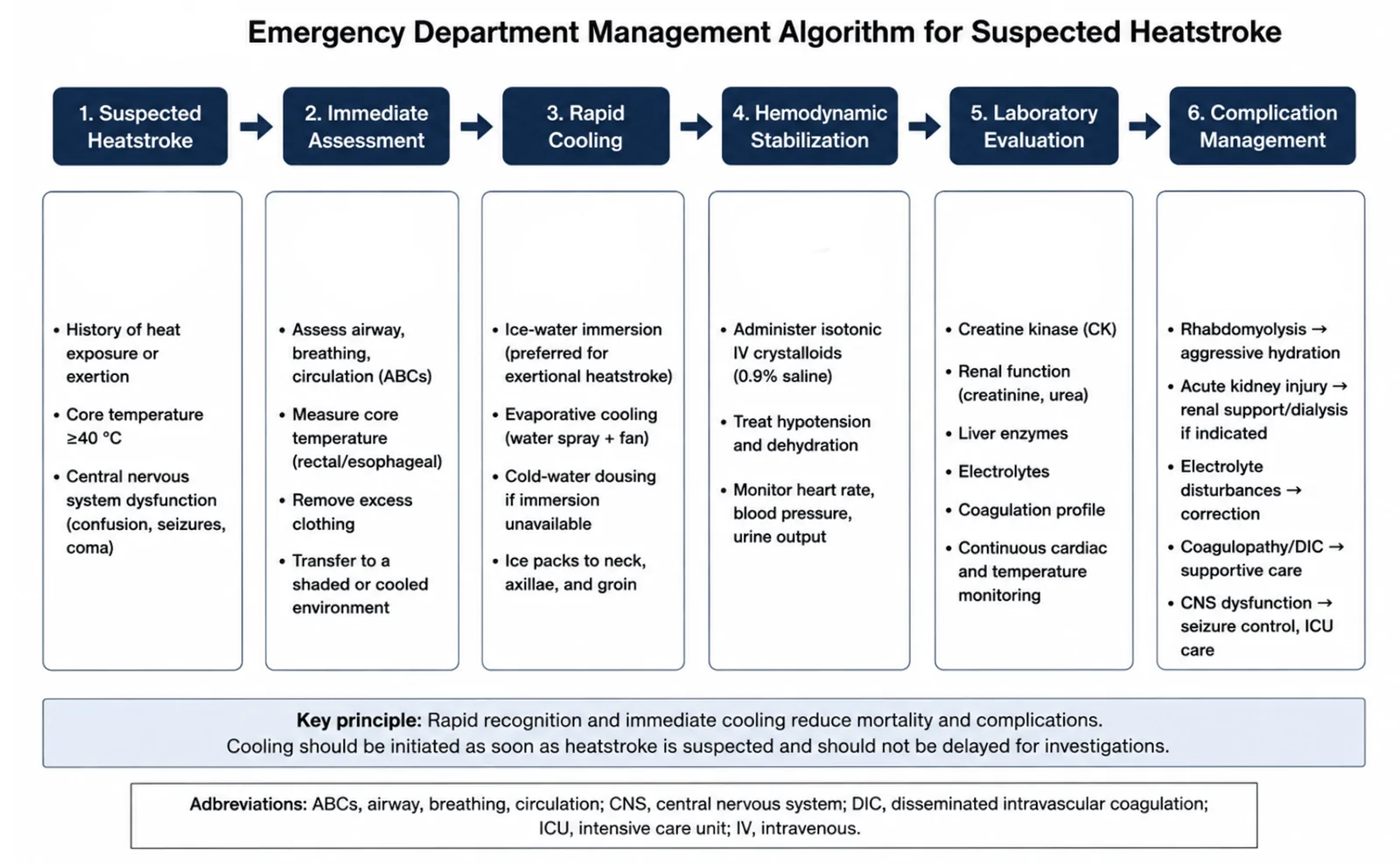
Recommended management algorithm for patients presenting with suspected heat stroke, emphasizing rapid cooling, airway stabilization, and supportive care. Original figure created by the authors.

Figure 3 presents a stepwise approach to the management of heatstroke in the ED, emphasizing early recognition, immediate intervention, and prevention of secondary complications. The algorithm begins with the identification of suspected heatstroke based on a history of heat exposure, elevated core body temperature (≥40°C), and central nervous system dysfunction. Immediate actions focus on securing the airway, breathing, and circulation while avoiding delays in cooling. Rapid cooling is highlighted as the primary life-saving intervention, using ice-water immersion, evaporative cooling, or alternative methods when necessary. Subsequent steps include hemodynamic stabilization with intravenous isotonic fluids, comprehensive laboratory evaluation with continuous monitoring, and early identification and treatment of complications such as rhabdomyolysis, acute kidney injury, electrolyte disturbances, coagulopathy, and neurological dysfunction. The figure underscores the time-sensitive, protocol-driven nature of heatstroke management in emergency settings.

In the Gulf region, special considerations arise in resource-limited or outdoor settings, where immediate access to emergency care may be delayed. On-site cooling is a critical strategy for laborers, athletes, or pilgrims before transportation to healthcare facilities. Rapid deployment of portable cooling devices, shaded rest areas, and accessible hydration stations can reduce the severity of HRIs and improve outcomes. For workers in construction or industrial environments, heat action plans, including scheduled breaks, worker education, and continuous monitoring of ambient temperatures, are essential components of preventive and emergency management strategies. In mass gatherings such as Hajj, dedicated cooling stations and mobile medical units have been implemented successfully to mitigate heat-related morbidity and mortality [31].

Outcomes and complications 

Heat-related emergencies can result in a wide spectrum of outcomes, ranging from rapid recovery with minimal intervention to severe multiorgan dysfunction and death. Short-term outcomes in the acute phase often include multiorgan involvement, necessitating admission to an intensive care unit (ICU) for monitoring and support. Common organ systems affected include the central nervous system, kidneys, liver, and cardiovascular system. Acute complications such as rhabdomyolysis, electrolyte disturbances, acute kidney injury, hepatic dysfunction, and coagulopathy are frequently observed in patients presenting with severe heatstroke, particularly in exertional cases. Early recognition and timely management, especially rapid cooling and aggressive supportive therapy, are crucial to minimize the severity of these acute outcomes [21].

Long-term sequelae may develop in patients who experience delayed or inadequate treatment. Neurological impairments ranging from cognitive deficits to persistent encephalopathy have been documented following severe heatstroke, reflecting the sensitivity of the CNS to hyperthermia. Similarly, renal impairment may persist after acute kidney injury, especially in cases complicated by rhabdomyolysis or prolonged hypotension. Cardiovascular sequelae, although less common, may include arrhythmias or chronic cardiac dysfunction, particularly in patients with pre-existing comorbidities. These long-term complications underscore the need for follow-up care and rehabilitation in survivors of severe HRIs [32].

Following stabilization of the acute illness, comprehensive rehabilitation is essential to optimize recovery and minimize long-term disability in survivors of severe heatstroke. A multidisciplinary approach involving physical medicine and rehabilitation specialists, physical therapists, occupational therapists, speech and language therapists, and neuropsychologists may be required depending on the patient's residual deficits. Rehabilitation should focus on improving mobility, balance, coordination, muscle strength, cognitive function, and independence in activities of daily living (ADLs). Particular attention should be given to patients with persistent neurological sequelae, such as cerebellar ataxia, cognitive impairment, or motor dysfunction, as these complications may significantly affect functional outcomes and quality of life. Individualized rehabilitation programs, combined with regular follow-up assessments, facilitate monitoring of neurological and functional recovery, allowing therapeutic interventions to be tailored to the patient's evolving needs. Emerging evidence suggests that structured rehabilitation can lead to meaningful improvements in functional independence and long-term recovery following severe heatstroke, highlighting the importance of integrating rehabilitation into the continuum of care for these patients [33].

Mortality rates associated with heatstroke vary depending on patient demographics, environmental context, and the timeliness of interventions. Globally, mortality for untreated heatstroke can exceed 50-80%, whereas aggressive cooling and supportive care can reduce mortality to 10-20%. In Gulf countries, reported mortality rates are variable due to differences in healthcare access, population characteristics, and environmental exposures, but several studies suggest rates comparable to global data when rapid cooling is implemented. However, among high-risk groups, such as elderly individuals, migrant laborers, and pilgrims exposed to extreme heat, mortality remains significant, particularly when interventions are delayed or unavailable [26].

Several predictors of poor outcomes have been identified in both Gulf and global populations. Delayed initiation of cooling is the single most critical determinant of morbidity and mortality, emphasizing the importance of immediate recognition and intervention. Underlying comorbidities, such as cardiovascular disease, diabetes, or chronic kidney disease, exacerbate vulnerability to heat-related complications. Exertional heatstroke, characterized by rapid onset and severe hyperthermia, is associated with higher rates of rhabdomyolysis, renal injury, and ICU admission compared to classic heatstroke. Additional factors influencing outcomes include extreme environmental conditions, inadequate hydration, and occupational or situational barriers that impede early access to care. Recognizing these predictors is essential for triaging high-risk patients and implementing targeted preventive and therapeutic measures in both clinical and field settings [31].

Discussion 

This literature review highlights the growing burden of heat-related emergencies in the Gulf region and underscores the profound impact of extreme climatic conditions on human health. As demonstrated by the preceding epidemiological and clinical evidence, the climatic conditions of the Gulf region now routinely exceed human thermoregulatory capacity, transforming HRI into a persistent health threat. The findings of this review demonstrate that HRIs in the Gulf are no longer sporadic or seasonal events, but rather a persistent public health challenge with significant clinical, occupational, and socioeconomic implications.

Compared with temperate regions such as Europe and North America, heat-related emergencies in the Gulf region are distinguished by the intensity, duration, and humidity of heat exposure. While heat illness in non-Gulf settings is often associated with short-lived heatwaves, the Gulf experiences prolonged periods of extreme heat lasting several months, resulting in sustained physiological stress and cumulative risk. In addition, higher ambient humidity and wet-bulb temperatures in Gulf countries markedly impair evaporative cooling, increasing the likelihood of severe illness even at lower levels of physical exertion [3].

Unlike many high-income temperate countries, the Gulf also has a disproportionately large population of migrant outdoor workers engaged in physically demanding labor, further amplifying exposure risk. These climatic and occupational differences help explain the higher baseline incidence of heat-related emergencies in the region and underscore the need for prevention strategies that extend beyond models developed in cooler or less humid climates [6].

Epidemiological evidence consistently shows a marked increase in heat exhaustion and heatstroke cases during the summer months across Gulf countries, particularly from May to September. The concurrence of extreme temperatures and high wet-bulb values is especially relevant, as these conditions severely impair evaporative cooling and substantially increase physiological heat stress. Saudi Arabia, the UAE, Qatar, and Kuwait report some of the highest rates of heat-related ED presentations globally, a trend that is likely to intensify as climate change accelerates. Importantly, these patterns mirror global observations but are amplified in the Gulf due to the region's climatic extremes and prolonged exposure periods.

A central theme emerging from this review is the disproportionate burden of heat-related emergencies among migrant and outdoor workers. Construction workers, oil-field laborers, delivery personnel, and other outdoor occupations are consistently overrepresented in heat-related hospitalizations. Exertional heatstroke is highly prevalent in this population due to the combination of intense physical activity, prolonged exposure to extreme heat, and limited access to hydration and cooling. These findings highlight the intersection between environmental risk and occupational vulnerability, emphasizing the need for stronger enforcement of heat-mitigation policies and workplace protections [34].

Mass gatherings and high-intensity events, particularly the Hajj pilgrimage, further compound heat-related risks in the region. The recent overlap of Hajj with the summer season has exposed millions of pilgrims to extreme temperatures, resulting in recurrent increases in heat-related morbidity and mortality. In response, Saudi Arabia has implemented a comprehensive heatwave mitigation strategy that extends beyond traditional medical preparedness. Measures include expanding shaded areas and cooling infrastructure, installing water misting systems and cooling stations, increasing the availability of drinking water, issuing heat-risk alerts and public awareness campaigns, optimizing crowd movement to reduce congestion, and strengthening emergency medical services with rapid identification and treatment of HRIs. Despite these substantial public health interventions, heat illness remains a significant challenge during Hajj, particularly as climate change continues to increase the frequency and intensity of extreme heat events. These experiences highlight the importance of integrating environmental modifications, public health planning, and emergency medical preparedness to reduce heat-related morbidity during large-scale mass gatherings [11,35,36].

From a pathophysiological perspective, this review reinforces that heat illness represents a continuum, progressing from mild heat exhaustion to life-threatening heatstroke. Once thermoregulatory mechanisms fail, hyperthermia initiates a cascade of cellular injury, systemic inflammation, endothelial dysfunction, and coagulopathy, ultimately leading to multiorgan failure. The overlap between heatstroke and sepsis-like physiology is particularly notable and has important implications for diagnosis and management in emergency settings. In the Gulf context, exertional heatstroke is especially prominent, with higher rates of rhabdomyolysis and acute kidney injury, reflecting the unique occupational and environmental exposures in the region [37].

Diagnostic challenges remain a significant barrier to optimal care. Delayed presentation to healthcare facilities is common, particularly among migrant workers, due to factors such as limited health literacy, language barriers, fear of job repercussions, and restricted access to medical services. In EDs, high patient volumes during peak summer months can further delay diagnosis and initiation of cooling. These challenges highlight the importance of frontline education, standardized diagnostic protocols, and the availability of rapid cooling methods both in prehospital and hospital settings [38].

Management strategies consistently emphasize immediate cooling as the most critical determinant of outcome. Evidence shows that rapid reduction of core body temperature dramatically reduces mortality and limits organ damage. However, access to effective cooling methods such as ice-water immersion or evaporative cooling varies widely across settings in the Gulf. Resource-limited or outdoor environments remain particularly vulnerable, reinforcing the need for on-site cooling measures, early recognition, and rapid referral systems. The integration of occupational health strategies with emergency medical care is therefore essential in reducing the severity of heat-related emergencies [12].

Although Gulf countries have implemented a range of preventive measures, including midday work bans, occupational heat regulations, and public cooling initiatives, evidence suggests that these interventions may be insufficient under intensifying climate conditions. Studies from the region demonstrate that heat stress indices frequently exceed safe thresholds even outside restricted working hours, indicating that current policies may not adequately reflect physiological risk in extreme environments. Moreover, variability in enforcement, limited worker awareness, and productivity-driven work practices can undermine the effectiveness of existing regulations [39].

Outcomes following heatstroke range from complete recovery to severe short- and long-term complications. Acute organ failure, ICU admission, and high mortality rates remain significant concerns, particularly in cases with delayed cooling or pre-existing comorbidities. Long-term sequelae, including persistent neurological impairment and chronic kidney disease, further contribute to the human and economic burden of heat illness. Predictors of poor outcomes identified in this review, such as delayed treatment, exertional heatstroke, and underlying chronic disease, should inform both clinical triage and preventive strategies [26].

Collectively, the findings of this review emphasize that heat-related emergencies in the Gulf are a multifactorial and escalating health threat driven by climate change, occupational exposure, and systemic vulnerabilities. Addressing this challenge requires a coordinated approach that integrates public health policy, occupational safety regulations, emergency preparedness, and climate adaptation strategies. Enhanced surveillance systems, standardized management protocols, and targeted interventions for high-risk populations are critical to mitigating the growing impact of extreme heat on health in the Gulf region.

Strengths and limitations 

This review has several notable strengths, including its region-specific focus on the GCC countries, which experience some of the most extreme heat conditions globally yet remain underrepresented in heat-health literature. By integrating epidemiological evidence with environmental and occupational risk factors, pathophysiological mechanisms, clinical presentation, ED management strategies, and outcomes, the paper provides a comprehensive and clinically relevant synthesis of heat-related emergencies within the unique climatic and sociocultural context of the Gulf. The use of multiple electronic databases enhanced the breadth of the literature captured, and the emphasis on vulnerable populations such as migrant workers and pilgrims increases the public health relevance of the findings. However, this review has several limitations. The available evidence was heterogeneous in terms of study design, populations, outcome definitions, and reported measures, limiting direct comparison across studies. Most included studies were observational or retrospective, reducing the strength of causal inferences and increasing the risk of reporting bias and residual confounding. In addition, the lack of standardized heat-illness surveillance systems and uniform diagnostic criteria across GCC countries limits the accuracy and comparability of epidemiological data. Finally, restricting the review to English-language, peer-reviewed publications may have excluded relevant regional evidence, introducing publication bias. These limitations suggest that the findings should be interpreted as a qualitative synthesis of the current evidence rather than definitive estimates of disease burden or intervention effectiveness.

## Conclusions

Heat-related emergencies in the Gulf region represent a critical and escalating health challenge that will intensify as climate extremes worsen. This review consolidates current evidence on the epidemiology, mechanisms, clinical implications, and management of heat illness within a uniquely high-risk environmental and occupational context, highlighting substantial gaps in surveillance, prevention, and research across GCC countries. The findings underscore the need for standardized regional reporting systems, improved early recognition and rapid cooling capabilities, and stronger integration of occupational and emergency care frameworks. Future research should prioritize multicenter prospective studies, standardized diagnostic criteria, evaluation of region-specific prevention strategies, and assessment of long-term functional and neurological outcomes among heatstroke survivors. From a policy perspective, strengthening occupational heat-protection regulations, implementing comprehensive heat-health warning systems, and expanding public education initiatives are essential to reducing heat-related morbidity and mortality. In clinical practice, the development of standardized emergency management protocols, regular healthcare provider training, and improved access to evidence-based cooling techniques and post-discharge rehabilitation services will be critical to enhancing patient outcomes and ensuring that healthcare systems across the GCC are equipped to respond effectively to the growing burden of extreme heat.

## References

[1] Pal JS, Eltahir EAB (2016). Future temperature in southwest Asia projected to exceed a threshold for human adaptability. Nature Climate Change.

[2] Raymond C, Matthews T, Horton RM (2020). The emergence of heat and humidity too severe for human tolerance. Sci Adv.

[3] Teyton A, Bailey J, Luo E, Ajaj R, Raymond C, Tuholske C, Benmarhnia T (2025). Overheated and understudied: A scoping review of heat-related health impacts in the Arabian Peninsula. Geohealth.

[4] Amoadu M, Ansah EW, Sarfo JO, Hormenu T (2023). Impact of climate change and heat stress on workers’ health and productivity: a scoping review. J Clim Change Health.

[5] Ahmed HO, Bindekhain JA, Alshuweihi MI, Yunis MA, Matar NR (2020). Assessment of thermal exposure level among construction workers in UAE using WBGT, HSI and TWL indices. Ind Health.

[6] Alahmad B, Al-Hemoud A, Al-Bouwarthan M (2023). Extreme heat and work injuries in Kuwait's hot summers. Occup Environ Med.

[7] Almuzaini Y, Alamri F, Alabdullatif L, Khan A (2025). Critical determinants of morbidity and adverse outcomes during the Hajj using the Haddon matrix and the combined model. Sci Rep.

[8] Aljumaan M, Alhawaj F, Alkhalifa S (2019). Awareness of heat-related illnesses in population of Saudi Arabia. Egypt J Hosp Med.

[9] Casa DJ, Armstrong LE, Ganio MS, Yeargin SW (2005). Exertional heat stroke in competitive athletes. Curr Sports Med Rep.

[10] Leyk D, Hoitz J, Becker C, Glitz KJ, Nestler K, Piekarski C (2019). Health risks and interventions in exertional heat stress. Dtsch Arztebl Int.

[11] Yezli S, Ehaideb S, Yassin Y, Alotaibi B, Bouchama A (2024). Escalating climate-related health risks for Hajj pilgrims to Mecca. J Travel Med.

[12] Narayanan A, Bell J (2024). Narayanan A, Bell J. UAE’s sweltering summer heat takes its toll on health facilities, workers and pets. https://english.alarabiya.net/infocus/2024/07/23/uae-s-sweltering-summer-heat-takes-its-toll-on-health-facilities-workers-and-pets.

[13] Alahmad B (2021). Alahmad B. Migrant workers bear the brunt of extreme heat in Kuwait. https://www.who.int/news-room/feature-stories/detail/migrant-workers-bear-brunt-extreme-heat-kuwait.

[14] Cramer MN, Gagnon D, Laitano O, Crandall CG (2022). Human temperature regulation under heat stress in health, disease, and injury. Physiol Rev.

[15] Dasari HP, Desamsetti S, Langodan S, Viswanadhapalli Y, Hoteit I (2021). Analysis of outdoor thermal discomfort over the Kingdom of Saudi Arabia. Geohealth.

[16] AlKahlout BH, Shariff M, Shaikh M, Jacob N, Hashim T (2017). Heat exhaustion in the emergency department: should not be confused with other forms of heat-related illness. Mediterr J Emerg Med.

[17] Al-Bouwarthan M, Quinn MM, Kriebel D, Wegman DH (2019). Assessment of heat stress exposure among construction workers in the hot desert climate of Saudi Arabia. Ann Work Expo Health.

[18] Abulibdeh A (2021). Analysis of urban heat island characteristics and mitigation strategies for eight arid and semi-arid gulf region cities. Environ Earth Sci.

[19] Almuzaini Y, Alburayh M, Alahmari A, Alamri F, Sabbagh AY, Alsalamah M, Khan A (2022). Mitigation strategies for heat-related illness during mass gatherings: Hajj experience. Front Public Health.

[20] Al-ajmi FF, Loveday DL, Bedwell KH, Havenith G (2008). Thermal insulation and clothing area factors of typical Arabian Gulf clothing ensembles for males and females: measurements using thermal manikins. Appl Ergon.

[21] Yip K, Tanen D (2025). Yip K, Tanen D. Overview of heat illness. MSD Manual Professional Edition. https://www.msdmanuals.com/professional/injuries-poisoning/heat-illness/overview-of-heat-illness.

[22] Gardner J, Caley L, Poche M, Trammell S (2024). Heat-related illness. Nursing.

[23] (2026). Heat exhaustion. https://www.mayoclinic.org/diseases-conditions/heat-exhaustion/symptoms-causes/syc-20373250.

[24] Iba T, Connors JM, Levi M, Levy JH (2022). Heatstroke-induced coagulopathy: biomarkers, mechanistic insights, and patient management. EClinicalMedicine.

[25] Iba T, Kondo Y, Maier CL, Helms J, Ferrer R, Levy JH (2025). Impact of hyper- and hypothermia on cellular and whole-body physiology. J Intensive Care.

[26] Adnan Bukhari H (2023). A systematic review on outcomes of patients with heatstroke and heat exhaustion. Open Access Emerg Med.

[27] Bouchama A, Abuyassin B, Lehe C, Laitano O, Jay O, O'Connor FG, Leon LR (2022). Classic and exertional heatstroke. Nat Rev Dis Primers.

[28] Garcia CK, Renteria LI, Leite-Santos G, Leon LR, Laitano O (2022). Exertional heat stroke: pathophysiology and risk factors. BMJ Med.

[29] Heat Exhaustion and Heat Stroke Are Too Hot (2023). Cleveland Clinic Health Essential. Heat exhaustion and heat stroke are too hot to handle on your own. https://health.clevelandclinic.org/heat-exhaustion-vs-heat-stroke.

[30] Savioli G, Zanza C, Longhitano Y (2022). Heat-related illness in emergency and critical care: recommendations for recognition and management with medico-legal considerations. Biomedicines.

[31] (2024). World Health Organization. Heat and health. https://www.who.int/news-room/fact-sheets/detail/climate-change-heat-and-health.

[32] Yezli S, Yassin Y, Ghallab S, Abdullah M, Abuyassin B, Vishwakarma R, Bouchama A (2023). Classic heat stroke in a desert climate: a systematic review of 2632 cases. J Intern Med.

[33] Seki M, Maeda T, Mizushima R, Kasai F (2025). Exploring occupational therapy interventions for heatstroke in emergency and critical care settings: a retrospective study. Cureus.

[34] van Selm L, Williams S, de'Donato F (2025). Occupational heat stress among migrant and ethnic minority outdoor workers: a scoping review. Curr Environ Health Rep.

[35] Nashwan AJ, Aldosari N, Hendy A (2024). Hajj 2024 heatwave: addressing health risks and safety. Lancet.

[36] Trujillo MH, Fragachán G C (2011). Rhabdomyolysis and acute kidney injury due to severe heat stroke. Case Rep Crit Care.

[37] Aldosari N, Yousaf N, Nashwan AJ (2026). Saudi Arabia's heatwave mitigation strategy during the Hajj. East Mediterr Health J.

[38] Khanal SK (2025). Language barriers and their consequences in healthcare: a qualitative case study of Nepali migrants in Finland. BMC Health Serv Res.

[39] Nazneen S, Choi SD, Ibarra-Mejia G (2025). Extreme heat exposure in the construction industry: a scoping review on risk factors and heat-related health consequences. Int J Environ Res Public Health.

